# Population Dynamics Among six Major Groups of the *Oryza rufipogon* Species Complex, Wild Relative of Cultivated Asian Rice

**DOI:** 10.1186/s12284-016-0119-0

**Published:** 2016-10-12

**Authors:** HyunJung Kim, Janelle Jung, Namrata Singh, Anthony Greenberg, Jeff J. Doyle, Wricha Tyagi, Jong-Wook Chung, Jennifer Kimball, Ruaraidh Sackville Hamilton, Susan R. McCouch

**Affiliations:** 1 Section of Plant Breeding and Genetics, School of Integrative Plant Science, Cornell University, 162 Emerson Hall, Ithaca, NY 14853 USA; 2 TT Chang Genetics Resource Center and International Rice Genebank, International Rice Research Institute, Los Baños, Laguna Philippines; 3 Present Address: Department of Plant Sciences, University of California, Davis, CA 95616 USA; 4 Present Address: School of Crop Improvement, College of PG Studies, Central Agricultural University, Umroi Road, Umiam, Meghalaya India; 5 Present Address: Department of Industrial Plant Science and Technology, Chungbuk National University, Cheongju, Chungubk 28644 Republic of Korea; 6 Present Address: Department of Crop Science, North Carolina State University, Raleigh, NC 27695-762 USA

**Keywords:** Population Structure, Domestication, Annual-Perennial, Chloroplast Diversity, Phylogeography

## Abstract

**Background:**

Understanding population structure of the wild progenitor of Asian cultivated rice (*O. sativa*), the *Oryza rufipogon* species complex (*ORSC*), is of interest to plant breeders and contributes to our understanding of rice domestication. A collection of 286 diverse *ORSC* accessions was evaluated for nuclear variation using genotyping-by-sequencing (113,739 SNPs) and for chloroplast variation using Sanger sequencing (25 polymorphic sites).

**Results:**

Six wild subpopulations were identified, with 25 % of accessions classified as admixed. Three of the wild groups were genetically and geographically closely related to the *O. sativa* subpopulations, *indica*, *aus* and *japonica,* and carried *O. sativa* introgressions; the other three wild groups were genetically divergent, had unique chloroplast haplotypes, and were located at the geographical extremes of the species range. The genetic subpopulations were significantly correlated (*r* ^2^ = 0.562) with traditional species designations, *O. rufipogon* (perennial) and *O. nivara* (annual), differentiated based on morphology and life history. A wild diversity panel of 95 purified (inbred) accessions was developed for future genetic studies.

**Conclusions:**

Our results suggest that the cultivated *aus* subpopulation is most closely related to an annual wild relative, *japonica* to a perennial wild relative, and *indica* to an admixed population of diverse annual and perennial wild ancestors. Gene flow between *ORSC* and *O. sativa* is common in regions where rice is cultivated, threatening the identity and diversity of wild *ORSC* populations. The three geographically isolated *ORSC* populations harbor variation rarely seen in cultivated rice and provide a unique window into the genetic composition of ancient rice subpopulations.

**Electronic supplementary material:**

The online version of this article (doi:10.1186/s12284-016-0119-0) contains supplementary material, which is available to authorized users.

## Background

The *Oryza rufipogon* species complex (*ORSC*) is the wild progenitor of Asian cultivated rice, *O. sativa* (Oka 1988; Barbier et al. 1991; Khush 1997), a crop that provides staple food for three billion people (Elert 2014). Both the *ORSC* and *O. sativa* are widely distributed across South, Southeast and Eastern Asia, but the wild stands exist mostly as small, isolated populations, adjoining or intermingling with cultivated fields (Vaughan et al. 2003). As such, wild stands are threatened by habitat destruction, admixture with *O. sativa,* and genetic erosion (Song et al. 2005). Seeds from thousands of crop wild relatives have been collected and preserved in gene banks around the world (Plucknett et al. 1983; Tanksley and McCouch 1997; Meilleur and Hodgkin 2004). These collections contribute to the conservation of natural variation, provide the foundation for biological research and insights into the domestication process, and they offer a genetically tractable source of novel variation for breeding (Brar and Singh 2011; McCouch et al. 2013). Yet little has been done to characterize them genetically or phenotypically. The lack of information makes it difficult to focus conservation and research efforts, or to utilize these crop wild relatives effectively for variety improvement (Gepts 2006, McCouch et al. 2012).

Historically, the species found within the *ORSC* are classified as either perennial (*O. rufipogon*) or annual (*O. nivara*), based on morphology, life/mating habit, and the ecological habitat in which they are found. The perennial form, *O. rufipogon*, is reportedly photoperiod sensitive and cross-pollinated; it is aquatic and found in areas with year-round standing water, such as swamps, river beds, and marshes. In contrast, *O. nivara* is considered to be annual, upright, photoperiod insensitive, and predominantly self-fertilized; it is found in seasonally wet habitats such as lake shores and river banks, which undergo periodic flooding with the monsoon rains (Barbier 1989; Li et al. 2006; Vaughan et al. 2008). A third designation, *Oryza spontanea,* is a mistaken contraction of *O. sativa* L. f. *spontanea* Roschev which refers to accessions derived from hybrids between *O. sativa* X *O. nivara* or *O. rufipogon* (Morishima et al. 1961; Chang 1976; Vaughan et al. 2001).

Previous studies have sought to interpret the genetic and geographical relationships among accessions in the *ORSC*, but differences in size of collections, geographical representation of germplasm, and/or marker coverage have led to different conclusions (Wang et al. 1992; Cheng et al. 2003; Londo et al. 2006; Molina et al. 2011; Huang et al. 2012a; Huang et al. 2012b; Banaticla-Hilario et al. 2013; Gross and Zhao 2014). In this study, we evaluate a panel of diverse *ORSC* accessions collected from 15 countries, including 56 accessions that overlap with previous reports, using genotyping-by-sequencing (GBS) and indel analysis for nuclear DNA, and Sanger sequencing for chloroplast DNA to: 1) characterize the population structure of the *ORSC*, 2) determine the relationship between the subpopulations of the *ORSC* and *O. sativa,* 3) elucidate the relationship between *ORSC* population structure, geographical distribution, annual-perennial life habit (based on traditional species designations), and archaeo-botanical history, and 4) select a subset of diverse accessions as the basis for developing an immortal wild diversity panel for future genetic studies.

## Results and Discussion

### Population Structure and Geographical Distribution of the *ORSC*

**Fig. 1:**
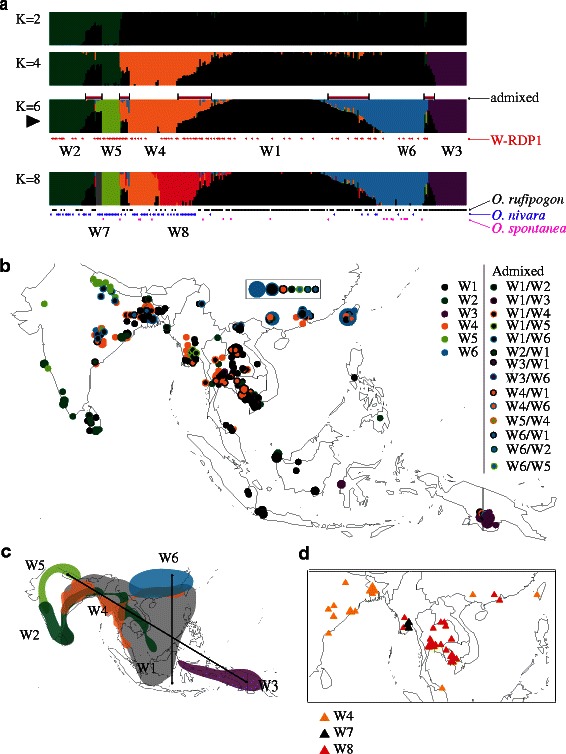
Population structure in the *ORSC*. **a** *fastStructure* analysis for 286 *ORSC* samples based on 113,739 SNPs where *black arrow* indicates optimal number of populations at *K* = 6 (see also Additional file 3: Figure S2A); admixed accessions sharing <75 % ancestry with any one subpopulation are highlighted by *red rectangles* above *K* = 6 panel; wild group numbers, W1-W6, correspond to order of divergence (as shown in Additional file 3: Figure S2A); accessions included in the Wild Rice Diversity Panel (Wild RDP, *n* = 95) indicated as red stars under *K* = 6 panel; traditional species designations, *O. rufipogon* (perennial), *O. nivara* (annual), and *O. spontanea* indicated by *black*, *blue* and *pink stars*, respectively, under *K* = 8 panel. **b** Geographical map showing distribution of samples from each subpopulation group where circle size corresponds to number of samples; fill color indicates subpopulation designation (*K* = 6); For admixed accessions, the first mentioned subpopulation represents the major proportion of ancestry; Chinese accessions lacking location detail indicated in closed rectangle; further detail provided in Additional file 1: Table S1A. **c** Simplified geographical map showing regional distribution of six subpopulation groups (*K* = 6); **d** Detailed view of geographical distribution of subpopulation groups (*K* = 8) highlighting relationship between W4 and W8

A collection of 286 geographically and genetically diverse accessions from the *ORSC* (Additional file 1: Table S1) was genotyped using GBS to generate a dataset consisting of 113,739 SNPs. Model-based analysis using marginal likelihoods predicted the optimal number of subpopulations to be *K* = 6, though there was little difference between *K*-values of 5–9 (Fig. 1a; Additional file 2: Figure S1). Based on *fastStructure* results at *K* = 6, 25 % of the *ORSC* accessions were classified as admixed because they had less than 75 % shared ancestry with one of the major subpopulation groups (Additional file 1: Table S1). The subpopulations were identified based on the order in which they diverged from the original population group (W1) with increasing values of K, such that Wild Group 2 (W2) diverged at *K* = 2, W3 diverged at *K* = 3, etc. (Additional file 3: Figure S2A). When the Neighbor Joining (NJ) method was used to analyze the same data, results were largely consistent with the model-based analysis at *K* = 6 (Additional file 4: Figure S3).

To determine whether the subpopulation groups identified by *fastStructure* were associated with a nonrandom geographical distribution, we mapped them onto a geographical map of Asia (Fig. 1b) and used the Mantel test to evaluate isolation-by-distance. An east-west axis separated the two most geographically isolated populations, W5 (Nepal) and W3 (Papua New Guinea), while a north-south axis (straddling the Himalayan Mountains) separated W6 (China and Taiwan) from a majority of the W1, W4 and W5 subpopulations (SE Asia) (Fig. 1c). W1 was the most widely distributed subpopulation, with accessions geographically co-mingled with other groups across both continental and archipelagic SE Asia. Consistent with its broad geographical distribution, W1 was also the most admixed subpopulation; it shared ancestry with a majority (93 %) of individuals classified as admixed in this study (*n* = 71). W2 accessions were also widely distributed across South and SE Asia, but were the predominant group in southern India and Sri Lanka. W3 accessions were found only in the geographically isolated Papua New Guinea region and were not found on the mainland. W4 accessions were widely distributed across SE Asia, extending west into northern India and east into southern China and Taiwan. W5 accessions were mainly from Nepal and western India, and were closely related to W2. W6 accessions were the predominant group in eastern Asia, found mostly in China and Taiwan. Interestingly, of the 16 W1/W6 admixed accessions in our collection, seven were from China or northern Vietnam, and nine were collected in Myanmar, NE India or Bangladesh (Additional file 1: Table S1).

At higher *K*-values, the emergence of W7 and W8 brought greater geographical definition to the subpopulations identified in SE Asia (Fig. 1a and d). At *K* = 7, a cluster of four accessions, previously classified as W1/W5 admixtures, was identified as a subpopulation from Myanmar. At *K* = 8, approximately half of the previously identified W4 accessions along with some admixed W1/W4 accessions, clustered as a separate subpopulation in SE Asia, geographically well differentiated from the remaining W4 samples found in E. India and Bangladesh (Fig. 1d).

Using the Mantel test to determine whether genetic distance was significantly associated with geographical distance, we found a small but significant correlation for the ORSC as a whole (not including admixed samples) (*r* ^2^ = 0.10, *p* < 0.001) (Additional file 5: Figure S4A, B). When the Mantel test was run separately on W2, W3 and W5 accessions, the most geographically isolated and least admixed among the *ORSC*, the association between genetic and geographical distance was significantly greater (*r* ^2^ = 0.439, *p* < 0.0003), and contrasted sharply with test results in W1, W4 and W6 accessions, the most widely distributed and most highly admixed subpopulations of the *ORSC* (*r* ^2^ = 0.0531, *p* < 0.001) (Additional file 5: Figure S4 B and C, respectively).

### Genetic Relationship Between *O. rufipogon* and *O. sativa*

We next re-analyzed the *ORSC* samples along with 45 *O. sativa* control varieties using Bayesian clustering based on the 113,739-SNP dataset. At *K* = 6, the same *ORSC* subpopulation groups were observed as when the data were analyzed without the *O. sativa* samples, but the cultivated samples allowed us to identify wild populations that clustered with specific *O. sativa* subpopulations (Additional file 3: Figure S2A, B). At *K* = 5 or *K* = 6, the W1 population shared >75 % ancestry with *indica* (black) accessions, the W4 population with *aus* (orange) accessions, and the W6 population with *japonica* (blue) accessions (*temperate japonica, tropical japonica* and *aromatic*). In contrast, W2, W3 and W5 did not cluster with any of the cultivated groups. These data support the hypothesis that the *aus,* the *indica* and the *japonica* subpopulations of *O. sativa* evolved from genetically distinct *ORSC* lineages. Further, they underscore the finding that the *aus* subpopulation is distinct from both *indica* and *japonica* and represents one of three domestication foci for rice in Asia (Garris et al. 2005; Londo et al. 2006; Schatz et al. 2014; Civáň et al. 2015).

To further examine the relationships between the *ORSC* and *O. sativa,* we compared pairwise genetic distance (GD) and *Fst* values to determine the degree of genome-wide divergence between wild and cultivated groups. These comparisons supported the close relationship between W1 and the *indica* subpopulation, W4 and *aus*, and W6 and *japonica*, while W2, W3 and W5 were maximally differentiated from the *O. sativa* subpopulations (Additional file 6: Table S2).

**Fig. 2:**
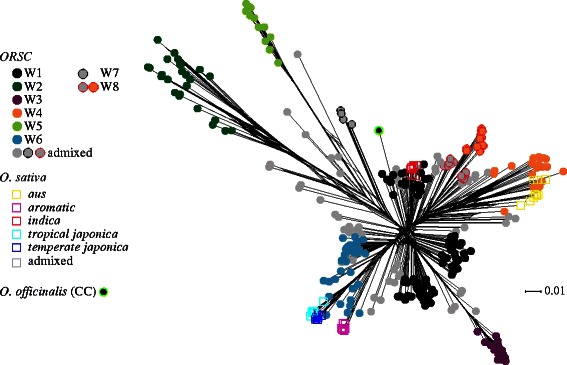
Phylogenetic network based on SNP data from the *ORSC* and *O. sativa* samples. *Circle color* corresponds to subpopulation identity as in Fig. 1b

When the NeighborNet method was used to analyze both wild and cultivated accessions (Fig. 2), results were largely consistent with the model-based analysis (Fig. 1a). At *K* = 6, *O. sativa, indica* (red) accessions were nested within one of the W1 clusters, *aus* accessions (yellow) emerged from one branch of the W4 cluster corresponding to samples from Bangladesh and India, the *temperate japonica, tropical japonica* and *aromatic* subpopulations (shades of blue and pink) emerged from the W6 group with long branch-lengths, and the three independent groups, W2, W3 and W5, were highly divergent based on long branch lengths with strong bootstrap support in the rooted NJ tree. W1 was found at the root position, and clustered with the *O. officinalis* (CC) outgroup, suggesting that the root position is among the W1 lineages. This interpretation was supported by the NJ dendrogram (Additional file 5: Figure S4) where nearly all groups in the *ORSC* had one or more W1 accessions as their sister group. Further, when the genetic divergence of *ORSC* subpopulations was compared, W1 had the lowest mean pairwise *Fst* and genetic distance (GD) (Additional file 6: Table S2B)*.*

The presence of the *O. sativa* samples in the analysis also revealed increased levels of admixture within the *OSRC*, particularly in the W1 (*indica-*like) and W6 (*japonica-*like) groups (Additional file 3: Figure S2B). While the cultivated *indica* and *japonica* subpopulations were clearly differentiated from each other, they each shared significant levels of ancestry with both W1 and W6 *ORSC* accessions. This suggested that complex patterns of migration had impacted the geographical distribution of both wild and cultivated groups, offering repeated opportunities for gene flow among and between them over the course of their history. If this were the case, we should be able to document regions of introgression from *O. sativa* in the *ORSC* genome, and vice versa.

To address this possibility, we surveyed the *ORSC* accessions for domestication-related seed and grain phenotypes where the genes underlying those phenotypes had been cloned and characterized, and then analyzed the genomic regions within and around the target genes in *ORSC* and *O. sativa* accessions to determine the origin of the DNA in accessions with wild-type or domestication-related phenotypes.

We focused our analysis on two domestication-related phenotypes that could be measured in seeds, hull color and pericarp color, to determine whether any of the *ORSC* accessions carried white hull and/or white pericarp, traits that were likely to have been inherited from *O. sativa*. Of the 157 accessions analyzed for these phenotypes, 22 (13 %) were found to carry one or both domestication traits (Additional file 7: Table S3). To determine whether the phenotypes were the result of domestication-related mutations, we analyzed DNA samples from a subset of the 22 ORSC accessions with white hull and/or white pericarp and a control set of 19 black hull, red pericarp accessions representing all wild subpopulation groups to determine whether they carried the wild type allele (conferring color) or the non-functional allele (associated with domestication) at the *BH4* gene (for hull color) and the *RC* gene (for pericarp color). Both genes had been previously cloned and the functional polymorphisms associated with the loss of color in *O. sativa* were determined to be a 22 bp deletion in *BH4* (Zhu et al. 2011) and a 14 bp deletion in *RC* (Sweeney et al. 2007).

PCR-based analysis of the 22 white hull and/or pericarp accessions and the set of 19 controls demonstrated that all but one of the *ORSC* accessions with white hull and/or white pericarp carried knock-out mutations associated with domestication. The exception was accession NSF_ID 474 where the seed stocks had white hull color, but the wild-type non-deletion *Bh4* allele was detected in the tissue sample used for genotyping. All but two of the *ORSC* accessions with black hull and red pericarp carried the wild type alleles; the exceptions being NSF-ID 540 and NSF-ID 460, both of which had seed stocks with black hulls but the individuals sampled for genotyping carried the 22 bp deletion *Bh4* allele (Additional file 1: Table S1)*.* The discrepancies are likely due to the heterogeneity of seed stocks, a common occurrence in *ORSC* accessions.

**Fig. 3:**
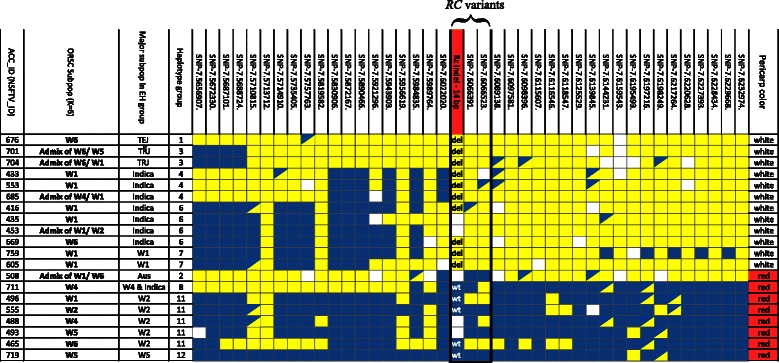
*RC* extended haplotypes for representative *ORSC* accessions. Extended haplotypes across a 576-kb window around the *RC* gene for 12 *white* pericarp and eight *red* pericarp *ORSC* accessions. NSF ID corresponds to accession number in Additional file 1: Table S1. The two SNPs and 14 bp indel within the *RC* gene are outlined in *black. Yellow* = cultivated allele; *blue* = wild type allele; *blue/yellow* = heterozygous; *white* = missing data. Note that all of the *white* pericarp accessions carry at least one cultivated allele at all three markers within the *RC* gene

To further confirm the origin of the domestication-related traits in *ORSC* accessions, we analyzed the SNP haplotypes surrounding the *RC* gene using ancestrally informative polymorphisms (Sweeney et al. 2007; Kovach et al. 2009; Lam et al. 2010; Takano-Kai et al., 2009). For this analysis, we included the same set of *ORSC* accessions that had been phenotyped and genotyped for the functional indel polymorphisms described above. We observed that the *ORSC* accessions carrying the knock-out (14 bp deletion) allele at *RC* carried an *O. sativa* extended haplotype around the *RC* locus while accessions carrying the wild type allele carried an *ORSC-*specific haplotype around *RC.* (Fig. 3, Additional file 8: Table S4, Additional File 9: Figure S5). This analysis supports the conclusion that the presence of domestication –related phenotypes in *ORSC* accessions are the result of gene flow and introgression from *O. sativa,* rather than standing variation in the wild.

It is noteworthy that *ORSC* accessions carrying domestication-related alleles belong to W1, W6 or were admixtures involving one or both of these subpopulations (Additional File 7: Table S3), consistent with the evidence that these two *ORSC* groups were most frequently admixed with *O. sativa*.

### Comparison of Subpopulation and Species Classification

Several different species names are used by gene banks to refer to accessions within the *ORSC*. When the six wild subpopulations identified in this study were analyzed in relation to the two primary species designations, *O. rufipogon* (perennial) and *O. nivara* (annual), we observed a significant correlation (*r* ^2^ = 0.562; Chi-square *p* < 0.0001) (Additional file 10: Table S5 and Fig. S6). Ninety one percent of W1, 100 % of W3 accessions, and 50 % of W6 accessions were classified as *O. rufipogon*, while a majority of W2 (56 %), W4 (64 %), and W5 (83 %) accessions were classified as *O. nivara* (Fig. 1a). Both species were found throughout mainland SE Asia, but *O. rufipogon* was predominant in the Indonesian archipelago (Additional file 11: Figure S7). The annual forms of W4 are closely related to *aus*, perennial forms of W6 are closely related to *japonica*, and the *indica* subpopulation shares ancestry with forms of W1 that show admixture with W4 on the one hand, and W6 on the other (Additional file 3: Figure S2B). This ancestral dichotomy, where both annual and perennial ancestors are recombined with W1 accessions, undoubtedly contributes to the high levels of diversity and broad adaptation observed within the *indica* subpopulation (Garris et al. 2005; Huang et al. 2012c).

This is the first report documenting the idea that the most recent wild ancestor of *indica* may have evolved as a complex derivative from divergent ancestral groups. Significant admixture is observed between W1 and W4 (annual) in India, Bangladesh and SE Asia, as well as between W1 and W6 (perennial) across SE Asia and into southern China. In this study, *ORSC* samples collected from Guangdong and Guangxi in southern China were related to both *indica* and *japonica*, while samples collected north of the Nanling mountains, in the central sub-tropical zone, were most closely related to *japonica*. The admixed nature of the ancestral W1 subpopulation is parallel to the scenario recently reported for barley (Pourkheirandish et al. 2015) but with the added dimension of coalescing annual and perennial life habits.

The 19 *O. spontanea* accessions shared >75 % ancestry with individuals in diverse subpopulations; 50 % of the samples were classified as W6, 22 % as W1, 17 % as W4, 11 % as W5, and one as an admixture (W1/W4) (Additional file 10: Table S5 and Figure S6). Because they did not cluster into a single genetic group, nor were they predominantly diagnosed as admixtures, we conclude that the species *O. spontanea* classification for these samples is not appropriate and should be dropped or reconsidered, given that it would be more informative to identify each sample in association with its most closely related wild subpopulation.

### Chloroplast Haplotype Analysis

**Fig. 4:**
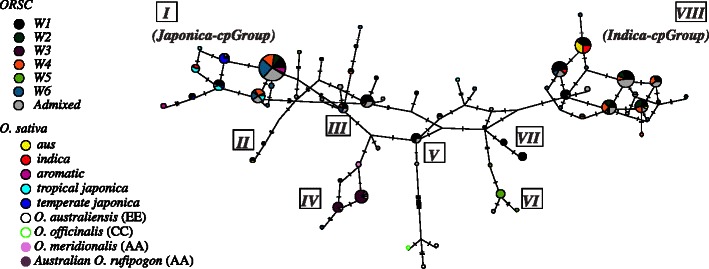
Chloroplast haplotype network. Haplotype network for the *ORSC* and *O. sativa* samples based on 25 chloroplast variants**;** single mutations indicated as hatches between haplotypes; chloroplast groups (*cpGroup*) *I* to *VIII* indicated in rectangles*;* size of nodes (circles) is proportional to haplotype frequency; colors indicate proportion of individuals from each subpopulation (based on GBS data at *K* = 6 in Fig. 1a) that carry the haplotype; *gray* indicates admixed accessions; for more detail, see Additional file 12: Figure S8 and Additional file 1: Table S1

**Fig. 5:**
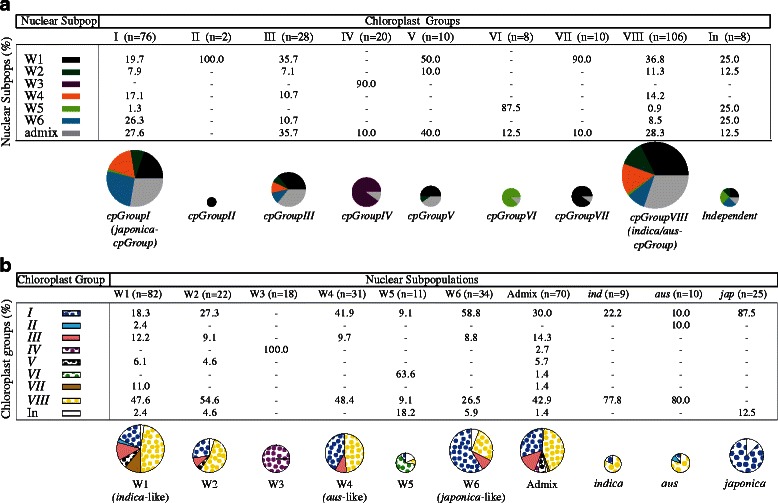
Chloroplast haplotype network. Incongruence between chloroplast group and nuclear subpopulation; **a** shows the frequency of nuclear subpopulations represented by individuals in each chloroplast haplotype group; **b** shows the frequency of chloroplast groups represented by individuals in each nuclear subpopulation

To further examine the extent and direction of gene flows among and between *ORSC* subpopulations and *O. sativa*, we assayed chloroplast sequence from five different regions of the rice chloroplast genome in 268 *ORSC* accessions, 44 *O. sativa* accessions, five AA genome wild accessions and three non-AA genome outgroups. Fifty-nine haplotypes were identified, and we generated a statistical parsimony haplotype network from these haplotypes, which clustered them into eight chloroplast groups (*cpGroup I*–*VIII*) (Figs. 4, 5; Additional file 1: Table S1). Not surprisingly, haplotypes from many of these groups were found in W1 individuals, consistent with nuclear data in suggesting that W1 comprises an ancestral, admixed, genetically diverse subpopulation; admixed individuals also shared haplotypes from different wild subpopulations. Excluding W1 and admixed individuals, there was good correspondence between chloroplast haplotype groups and subpopulations. *cpGroup IV* was unique to W3, and *cpGroup VI* was unique to W5 accessions. These chloroplast haplotypes lend support to the results of the *fastStructure* analyses and provide evidence of distinct maternal lineages in wild subpopulation groups. At the same time, several haplotype groups were shared by different wild and cultivated subpopulations, supporting the conclusion that both ancient and (in the case of cultivated accessions) more recent gene flow continue to dilute the once-distinctive gene pools (Fig. 5: note *cpGroups I, III,* and *VIII*).

Haplotypes of outgroups (*O. officinalis* (CC) and *O. australiensis* (EE)) were very distinct from those of the *ORSC.* The outgroup haplotypes joined the network at *cpGroup V*, a haplotype found almost exclusively in W1 and admixed individuals, further supporting the ancestral nature of the W1 group. The network had several loops; given the historically non-recombining nature of the chloroplast genome, loops are interpreted as being due to substitutional parallelisms and reversals rather than to recombination. This reticulate structure complicates interpretation of the network; however, outgroup rooting clearly split the network into two large groups strongly associated with the two major *O. sativa* varietal groups, *JAPONICA* (*tropical japonica, temperate japonica, aromatic*) and *INDICA* (*indica, aus*), referred to as *cpGroup I* (or the *JAPONICA-cpGroup*) and *cpGroup VIII* (or the *INDICA-cpGroup*), respectively. *cpGroup I* haplotypes were found in 87.5 % of cultivated *japonica* cultivars and 58.8 % of W6 accessions, the most closely related ancestral group, while *cpGroup VIII* haplotypes were found in 77.8 % of cultivated *indica,* 80 % of cultivated *aus* cultivars, and only 47.6 and 48.4 % of the related W1 and W4 accessions, respectively. The divergence between these two chloroplast groups is not as obvious in the *ORSC* accessions as it is in the *O. sativa* groups. This is consistent with the results of the Mantel test suggesting that geographical dispersion of *ORSC* populations and admixture with *O. sativa* (particularly for W1, W4 and W6) has eroded the genetic composition of the ancestral populations from which *O. sativa* was originally domesticated.

Along one path from the outgroup to the *JAPONICA-cpGroup I,* the first group of accessions to diverge was *cpGroup IV*, found primarily in the geographically isolated W3 accessions from Papua New Guinea and Australia and the closely related AA genome species, *O. meridionalis*. Along the alternative path toward *JAPONICA- cpGroup I*, the *cpGroup III* diverged; this group was most common in admixed and W1 individuals. In the other half of the network, along the path leading to the *INDICA-cpGroup VIII* were *cpGroups VI* and *VII*; haplotypes of the former group were found exclusively in individuals of subpopulation W5, from Nepal (colored light green), whereas haplotypes of the latter group were found only in W1 accessions (Fig. 4; Additional file 12: Figure S8)*.*

Seventy-eight percent % of individuals from the *O. sativa*, *indica* subpopulation and 90 % of individuals from the *aus* subpopulation carry haplotypes from *cpGroup VIII*, while 100 % of *japonica* individuals carry haplotypes from *cpGroup I*. This suggests the *aus* and *indica* subpopulations share a more recent maternal ancestor than either does with *japonica,* consistent with previous findings (Garris et al. 2005; Londo et al. 2006). Interestingly, the analysis also supports the conclusion that when intersubpopulation hybridization occurred between early domesticates, individuals from the *indica* and *aus* subpopulations were more likely to have served as the maternal parents, and *japonica* as a pollen donor.

We next examined specific chloroplast sequence polymorphisms that were shared between *ORSC* and *O. sativa* (Fig. 4; Additional file 13: Table S6A). One of the *indica/aus-*specific derived variants corresponds to a 69 bp deletion (#6) which is widely used to differentiate *japonica* (ancestral, non-deletion type) from *indica/aus* (derived, deletion type) in phylogenetic studies (Kanno et al. 1993; Garris et al. 2005). In addition to the 69 bp deletion, we discovered a single derived SNP located inside the indel (#7 at 8599 bp) that was found in non-deletion types, predominantly in *japonica* (“G”), while the ancestral SNP (“A”) was exclusively found in all out-groups and other AA genome species (Additional file 13: Table S6B). Within the *ORSC*, two geographically divergent subpopulations, W3 (from Papua New Guinea) and W5 (from Nepal) both harbored the “G” SNP within the non-deletion allele (at frequencies of 100 and 90.0 %, respectively), while the rest of the wild subpopulations collected across South and SE Asia and southern China, contained a mixture of all three chloroplast genotypes: the 69 bp non-deletion type with SNP-A, 69 bp non-deletion type with SNP-G, and the 69 bp deletion type.

The fact that chloroplast haplotype patterns are not identical to the nuclear genome groups in either wild or cultivated rice is not unexpected; rather it underscores the complex population dynamics in both the *ORSC* and *O. sativa*, where deep coalescence (incomplete lineage sorting) and recent hybridization (admixture) both play a role. Because these two processes produce the same signature of incongruence, it is difficult to disentangle them or to accurately interpret the timing of events that contribute to the patterns of diversity among and between populations.

### Development of Wild Rice Diversity Panel (W-RDP)

Based on these studies of nuclear and chloroplast variation, 95 *ORSC* accessions were selected to represent the major subpopulation groups as part of the Wild Rice Diversity Panel 1 (W-RDP1) (Fig. 1a; Additional file 1: Table S1). As the basis for replicated phenotypic evaluation and genome wide association mapping, a single individual from each accession was selfed for three generations to genetically purify the lines. Seed production in the greenhouse on these wild, shattering plants was very limited in the Ithaca environment, and with successive generations of inbreeding, there was a noticeable reduction in the quantity and quality of seed set on many of the plants, most notably those in the W3 subpopulation. The result was that none of the W3 individuals generated viable S_3_ seed. Nonetheless, we were able to generate S_3_ seed on a diverse collection of 95 *ORSC* accessions representing the W1, W2, W4, W5 and W6 subpopulations. These purified (self-pollinated) seed stocks represent a valuable genetic resource as the basis for future genetic studies in this crop wild ancestor.

### Evolutionary History and Population Dynamics

**Fig. 6:**
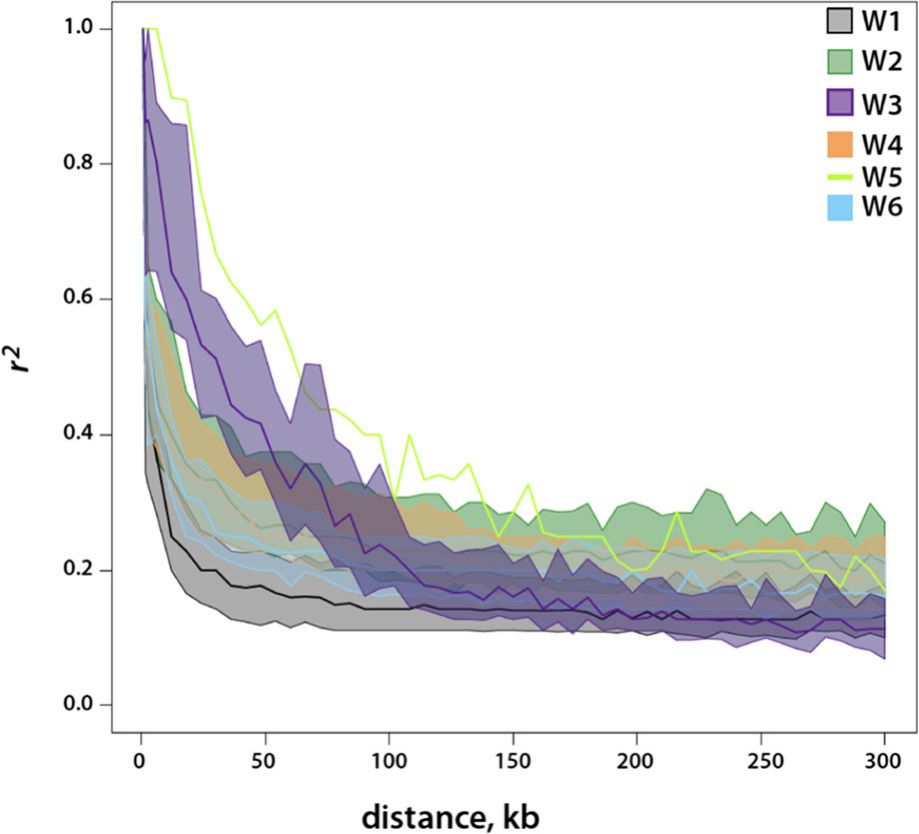
LD Decay for each Subpopulation (See also Additional file 15: Table S7)

To gain further insight into the evolutionary history and population dynamics of the wild subpopulations, we compared levels of nucleotide diversity (π) and linkage disequilibrium (LD) decay among groups. Of the wild accessions not closely related to any cultivars, W3 and W5 behave as expected for small isolated populations: their within-population diversity is low, (Additional file 14: Figure S9) and divergence from all other groups is high (Fig. 2; Additional file 6: Table S2B), likely due to a combination of genetic drift and local adaptation. However, these two populations are distinguished by their levels of LD (Fig. 6; Additional file 15: Table S7); the population from Papua New Guinea, W3, contains individuals that are exclusively classified as *O. rufipogon* using the traditional annual-perennial nomenclature system, and has relatively rapid LD decay, consistent with the out-crossing nature that is characteristic of most perennials, while W5 (mainly from Nepal) has >80 % of individuals classified as *O. nivara* and maintains LD over larger distances than any other subpopulation, in keeping with its predicted inbreeding habit.

Population W2 is unusual. It is the first group to be differentiated from W1 in *fastStructure* analysis, its level of nucleotide diversity (π) is high, (Additional file 14: Figure S9) yet it has extensive LD (Fig. 6; Additional file 15: Table S7). This suggests that while the effective population appears to be large, there is not much recombination among individuals. Similar to W5, W2 accessions are predominantly identified as *O. nivara,* which suggests a high level of self-pollination, but W2 is more widely distributed geographically, being abundant in eastern India and isolated parts of southern India and Sri Lanka. This raises interesting questions about the potential for the annual habit to have arisen multiple times in response to diverse climatic factors across a broad geographical range. We hypothesize that the high level of π, combined with the extensive LD observed in the W2 population may be the result of a rapid evolutionary process that favored survival of numerous geographically dispersed and genetically isolated populations that were independently able to transition to an annual, inbreeding habit in response to a dramatic change in climate, such as that which has been described as global warming at the end of the Pleistocene era (Fuller et al. 2010).

The W4 subgroup is also characterized by high estimate of π (similar to that of W2), but has rapid LD decay. It has a distinctive relationship with the *aus* subpopulation and is also predominantly comprised of *O. nivara* accessions, again suggesting a strong annual growth habit. W4 is distributed throughout Bangladesh, northern Myanmar and Eastern India (Khush 1997; Garris et al. 2005; Londo et al. 2006). Its deep subpopulation structure offers further evidence that the annual growth habit may have evolved multiple times from different ancestral populations. The W4 subgroup and its *aus* relatives are increasingly recognized as a source of unique, stress-tolerance traits of interest to plant breeders for developing new, climate-resilient rice varieties (Bin Rahman and Zhang 2016; Famoso et al. 2011; Schatz et al. 2014). With its unique geographic, genetic and ecological history, the cultivated *aus* subpopulation and its wild ancestors (W4) represent an underappreciated genetic resource.

W6 represents a group of *ORSC* accessions collected in China and Taiwan, the presumed center of domestication for the *japonica* subspecies of *O. sativa* (Londo et al. 2006; Kovach et al. 2009; Huang et al. 2012b). This group has low to intermediate levels of π and LD decay, consistent with its recent expansion into the temperate region in eastern Asia, the northern-most tip of the zone inhabited by the *ORSC*. Low diversity would be expected at the forefront of a range expansion or in isolated colonizing groups, as is the case for *temperate japonica*. Some wild diversity, particularly the ancestral populations from which the earliest *japonica* cultivars were domesticated, has surely also been lost as human civilization encroaches on its habitat (Song et al. 2005). In this study, W6 samples from southern China were more likely to share ancestry with W1 wild accessions than were samples from farther north, contributing to the loss of identity of the ancestral *japonica* gene pool (Wang et al. 2008).

Within the *ORSC*, W1 is a heterogeneous group that is at the center of the network of relationships (Fig. 2). It has the most diverse representation of chloroplast haplotypes (Fig. 5), the most rapid LD decay (Fig. 6), and is geographically the most widely distributed wild subpopulation (Fig. 1). It has hybridized extensively with several other groups to produce admixed individuals. The geographic distribution and genetic similarity of W1 to other wild and domestic populations suggest the possibility that it may be ancestral to the entire *ORSC*. Under this scenario, it is interesting to speculate how ecological, genetic, and climatic changes may have contributed to the differentiation of the other groups.

The surprising observation that W1 has only intermediate π (Additional file 14: Figure S9) suggests that, rather than being ancestral to the entire *ORSC,* it may actually be a product of secondary hybridization between an assortment of populations. A high level of admixture is characteristic of a majority of *ORSC* gene bank accessions. While exhibiting numerous “wild” phenotypic characteristics, these accessions also carry numerous “cultivated” alleles inherited from *O. sativa*, as demonstrated for hull and pericarp color in this study. The value of the W1 population for plant breeding is that it provides a wealth of novel allele combinations whereby the genome has been introgressed and recombined over many thousands of years. Due to its broad geographic and ecological distribution, this wild subpopulation has also been exposed to extensive natural and artificial selection, acquiring diverse forms of disease and insect resistance, abiotic stress tolerance, grain quality, and physiological characteristics that provide plant breeders with valuable allele complexes for adaptive breeding and variety development.

### Climate and Species Range

The current range of the *ORSC* extends across a northwest (W2 and W5) to southeast (W3) axis, with the subpopulations most closely affiliated with *O. sativa* (W1, W4, W6) bracketed by those extremes. (Fig. 1c). This observation is consistent with Fuller et al.’s (2010) hypothesized climate-based shifts in the ranges of ancestral wild rice habitat since the Pleistocene. This hypothesis asserts that 20,000 years ago, during the Last Glacial Maximum, wild rice populations were limited to wet tropical refugia such as Eastern India, Southern China, and continental Southeast Asia, which extended down into the then-interconnected northern Indonesian peninsula. Subsequent changes in climate, characterized by increased temperatures, a rise in atmospheric CO2, and periodic dry seasons followed by monsoon rainfalls helped to expand the range of the *ORSC* and alter the population dynamics. Increasing temperatures in the northern hemisphere would be predicted to support the expansion of wild rice populations northwards, consistent with the identification of the W6 subpopulation located as far north as the Yangtze River basin in China and the W5 subpopulation in the highlands of Nepal. The emerging monsoon climate with its long, hot, dry summers, particularly pronounced on the Indian subcontinent and across into SE Asia, would have selected for new, wild, annual forms of *O. nivara,* such as those observed in the dispersed W2 and W4 subpopulations in this study. In the southernmost ranges, rising sea levels would have inundated low-lying land bridges and created islands of reproductively isolated *ORSC* populations, consistent with the W3 subpopulation documented from Papua, New Guinea. Into this scenario of wild rice population dynamics, humans began to experiment with early domestication efforts, introducing an additional agent of change that contributed to population movement and helped to obfuscate the wild subpopulation structure that once existed across South and SE Asia. While our study detects the impact of these events, documented in the observed patterns of admixture, we make no claims as to the timing of population expansion because it is unclear how biases in calling SNPs from GBS data would affect the site frequency spectrum and thus obscure any demographic signal.

Geographically isolated *ORSC* populations provide a unique opportunity to document the genetic composition of ancient subpopulations of wild rice. In this study, we document an unusual case of a chloroplast haplotype shared between accessions of W3 (Papua, New Guinea), W5 (Nepal) and two outgroups, *O. officinalis* (CC-genome) and *O. australiensis* (EE-genome), suggesting the possibility that the geographically isolated W5 and W3 subpopulations may have radiated from a common ancestor at about the same time. Isolated populations such as these that survive in natural refugia are of great interest for genetic studies and pre-breeding applications in rice improvement because they are likely to harbor variation rarely seen in cultivated rice. They also warrant special conservation efforts because they are increasingly threatened by habitat destruction.

Research aimed at exploring the diversity and population structure of other *Oryza* species, particularly those native to Australia and New Guinea, is of interest to expand our understanding of both the AA genome and more distantly related *Oryza* relatives that exist in isolated populations in that part of the world (Waters et al. 2012; Sotowa et al. 2013). In this study we found an Australian accession of *O. rufipogon* corresponding to subpopulation W3 that shared a chloroplast haplotype with three *O. meridionalis* accessions, suggesting either shared ancestry or gene flow between the two species (Cai et al. 2008). Such findings can help clarify the evolutionary history of the *Oryza* genus.

Reports of admixed accessions being found far from the geographical regions occupied by their immediate ancestors support the idea that small subsets of the *ORSC* likely traveled (and continue to be moved) along with cultivated *O. sativa* in the form of mixed/contaminated seed lots through commercial trade and human migration. This, along with back-introgression from *O. sativa* to *ORSC* in farmer's fields, could explain the presence of such geographically unexpected admixed subpopulations. The fact that W1/W6 admixed accessions are found in eastern China and as far west as NE India is consistent with dissemination by humans and with genetic and archeological evidence documenting hybridization between *japonica* rice from Southern China and proto-*indica* rice in North India (Fuller 2011). In addition, there are several reports of key domestication traits being introgressed from domesticated *japonica* varieties into *indica* (Sweeney et al. 2007; Takano-Kai et al. 2009; Kovach et al. 2009; Yang et al. 2011). These observations suggest that humans have contributed to the complex hybridization and introgression patterns observed in the *ORSC* over thousands of years and across a wide geographical range. Further, in this study of the *ORSC,* we see that humans have left their mark not only on the populations they domesticated, but also on the wild relatives they left behind.

## Conclusions

Six wild subpopulations were identified in a collection of 286 diverse *ORSC* accessions originating from 15 countries. Three of the wild groups were genetically and geographically closely related to the three major *O. sativa* subpopulations, *indica*, *aus* and *japonica,* while three other wild groups were genetically divergent, each with unique chloroplast haplotypes. The three divergent wild subpopulations were located at the geographical extremes of the species range, while the wild relatives most closely related to *O. sativa* were located across S. Asia, continental SE Asia, and southern China and shared significant levels of admixture with each other and with *O. sativa*. Correlations between the *O. rufipogon* complex subpopulations defined based on molecular variation in this study and the two traditionally recognized species groups, *O. rufipogon* (perennial) and *O. nivara* (annual), classified based on morphology, mating habit, and ecological habitat, suggest that the cultivated *japonica* subpopulation derives from a perennial ancestor, the *aus* subpopulation from an annual wild relative, and that *indica* is the result of admixture between divergent annual and perennial wild ancestors. Our findings are consistent with the hypothesis that the annual habit likely arose multiple times in response to diverse climatic factors across a broad geographical range. Understanding the relationship between subpopulation structure, ecology and geography is crucial for breeding programs seeking to harness the wealth of natural variation that resides in crop wild relatives. As part of this study, we also developed a wild diversity panel consisting of 95 purified (inbred) accessions representing the range of variation in the *ORSC* complex as the basis for future genetic studies.

## Methods

### Germplasm

Seeds from 286 *ORSC* accessions were imported from the International Rice Germplasm Collection (IRGC; *n* = 283) at the International Rice Research Institute in the Philippines and from the National Institute of Genetics (*n* = 3) in Japan (Additional file 1: Table S1). Fifty accessions of *O. sativa* from the Rice Diversity Panel 1 (RDP1) (Eizenga et al. 2014) were used to evaluate the relationship between wild and cultivated rice (Additional file 1: Table S1)*.*

### Phenotyping

#### Hull and Pericarp Color Phenotyping

Hull and pericarp color were phenotyped on all 286 *ORSC* accessions grown out at the Guterman Bioclimatic Laboratory from 2006 to 2007. Hull color and pericarp color were evaluated on three seeds each, produced by three individuals from each accession. Hull color was annotated as black hull or light hull. Pericarp color was scored numerically: red pericarp = 1.0, white pericarp = 0 and pericarp color scores were then averaged across individuals to determine the accession mean. Only 157 of these 286 accessions with complete data for both hull and pericarp color were included in the final analysis.

### Genotyping

#### DNA Extraction

Young leaf tissue was collected from single plants for DNA extraction using a modified potassium acetate-SDS protocol (Dellaporta et al. 1983) and DNeasy Plant Mini Kit (Qiagen).

#### Genotyping-By-Sequencing

96-plex GBS libraries were prepared using the *Ape*KI restriction enzyme; libraries were sequenced using an Illumina HiSeq 2500 (Elshire et al. 2011). SNP calling and filtering was done using the Tassel 3 GBS Plugin (Glaubitz et al. 2014). The sequence tags were aligned to the Nipponbare reference genome (MSU v6) using Bowtie2 (Langmead and Salzberg 2012). A set of 113,739 SNPs with call rates greater than 50 % per SNP locus (average 72 %) and with Minor Allele Count (MAC) >3 well distributed across the 286 *ORSC* and 45 *O. sativa* genomes were used for analyses of wild materials (Additional file 1: Table S1A, B). More detailed information about Materials and Methods is provided as Additional file 16.

### Chloroplast Markers

Sequence information for four AA genome and two EE genome wild control accessions were selected from Genbank; three *O. meridionalis* (GU592208, JN005831, and NC_016927, AA genome) and one Australian *O. rufipogon* (JN005833, AA genome), and two *O. australiensis* (GU592209 and KJ830774, EE genome), (Additional file 1: Table S1C). Sequence data were aligned to the reference genome, NC_001320, implemented by Geneious v7.1.7.

A total of 36 sequence variants were selected from 4127 bp of concatenated chloroplast sequence representing five different regions in the *O. rufipogon, O. sativa, O. meridionalis, O. officinalis* and *O. australiensis.* Of these, 25 variants were polymorphic within *ORSC* (Additional file 13: Table S6A) and were selected for diversity analysis. Chloroplast sequence data were generated as described in Kim et al. (2014) (Kim et al. 2014).

### Data Analysis

#### Nuclear Data

##### Population Structure and Genetic Relationships

Population structure was investigated using *fastStructure* with a simple prior (Raj et al. 2014) and visualized in *distruct* (Rosenberg 2004). The range of optimal K (number of populations) values to be tested was determined based on model complexity using marginal likelihood and model components to explain the structure of the data. Genetic relationships were also investigated as a network using an unrooted Neighbor Joining (NJ) algorithm implemented in SplitsTree v4 (Huson and Bryant 2006) and a rooted NJ dendrogram with 100 bootstrap replicates in Geneious v7.1.7. Genomic diversity between individuals and subpopulations was visualized based on NJ genetic distance as a heatmap using the devtools package in R 3.0.1. The chi-square statistic, implemented by JMP Pro V10 (SAS Institute Inc.), was used to determine whether the subpopulation designations for the *ORSC* accessions based on GBS data corresponded to taxon names used by the IRGC.

##### Isolation by Distance

The relationship between geographical and genetic distance was analyzed based on Mantel’s test using Isolation By Distance v3.23 (Jensen et al. 2005) with 1000 randomization cycles. Nineteen accessions from China with unknown geographical location within the country were excluded from these analyses.

##### Calculation of Fst, π, and d

Pairwise *Fst* statistics among subpopulations were calculated based on the average value over non-overlapping sliding windows of 100 SNPs across the genome with 95 % empirical Confidence Interval (CI) (Weir and Cockerham 1984). Using the same 100 SNP windows, we calculated π and *d*. We enumerated the sequence differences between a given pair of DNA segments and calculated sequence differentiation using the Jukes-Cantor model (Li 1997). Genetic distances between population pairs and nucleotide diversity within populations were estimated based on Nei (1973). For estimates of within-population π for *ORSC* populations, we used the full set of 113,739 SNPs; for calculating each pairwise genetic distance, only polymorphic SNPs were used. To enable comparisons between different analyses, we estimated per-kb values of π and *d* by dividing the total value for a window by the reference map distance (in kb) between the first and last SNP.

##### Haplotype Analysis

Extended haplotypes (EHs) spanning a 580 kb region flanking the *RC* locus on chromosome 7 were constructed on 81 *ORSC* accessions from the Wild Rice Diversity Panel (W-RDP1) (Additional file 1: Table S1) and on 406 *O. sativa* accessions from RDP1 genotyped with the HDRA (McCouch et al. 2016). The HDRA carries a total of 1021 SNPs in the 580 kb region. SNPs with a MAF > 0.05 and < 3 % missing data were initially selected. SNPs were then filtered based on a frequency test; only SNPs with a significant frequency difference between *O. sativa* accessions with white pericarp and *ORSC* accessions with red pericarp (*P* value cutoff: 1.0e −05) were used. The final set of SNPs used to construct the haplotype map in Fig. 3 consisted of 40 SNPS (Additional file 17: Table S8).

##### Linkage Disequilibrium

Linkage disequilibrium (LD; estimated as *r* ^2^ between SNPs) within populations was calculated in 10 Megabase windows using Plink v1.9 (Purcell et al. 2007). We retained SNPs with no more that 30 % missing data and at least two individuals carrying the minor allele. Raw pairwise estimated were binned by distance range. We present the LD estimates as means within a bin. Because W5 was the smallest population (with 12 samples), we sub-sampled 12 accessions from the other groups 100 times each and re-ran the LD analyses to account for any effect of sample size on the *r* ^2^ statistic. Figure 6 thus shows a mean and 95 % confidence interval of LD decay rates for each population, with the exception of W5 which has not been sub-sampled and thus has only one value per distance bin.

#### PCR Analysis of *RC* and Bh4 Indel Polymorphisms

PCR primer pairs were designed to amplify a 236 bp region spanning the functional 14 bp indel of *RC* (Sweeney et al. 2006) and a 227 bp region spanning the functional 22 bp indel of *Bh4* (Zhu et al. 2011), with product sizes optimized for indel resolution via agarose gel electrophoresis*.* DNA was extracted from tissue of 41 *ORSC* accessions from McCouch lab W-RDP biobank samples. PCR was done with a Tm of 56 °C for the *BH4*-M22 primer set and 57 °C for the *Rc-1* primer set. Reactions were run out on a 5 % agarose gel for 3 h and scored. Primer sequences are as follows: *BH4*-M22F 3’-TCTGGTGCATAATCAGAATGG-5’; *BH4*-M22R 3’-TCGTGTATATGGCGACCTTG-5’; *Rc-1* F 3’-CTTGCCAGTTTCAGAGAAATCA-3’; *Rc-1* R 3’-CTCTTTCAGCACATGGTTGG-5’.

#### Chloroplast Data

A statistical parsimony haplotype network was generated for 268 *ORSC* accessions, 44 *O. sativa* accessions, five AA genome wild accessions and three non-AA genome outgroups, one *O. officinalis* (CC) and two *O. australiensis* (EE) (Additional file 1: Table S1A, B and C), based on chloroplast sequence information using TCS v1.21 (Clement et al. 2000), implemented by POPART (Leigh and Bryant 2015). Sequence data were aligned to the reference genome, NC_001320. Every polymorphism was given the same weight, under the assumption that each represented a single evolutionary event. Chloroplast groups were defined as a continuum of haplotypes at 97 % parsimony connection (Ray et al. 2013) and haplotypes not belonging to any cpGroup were considered independent haplotypes (*ln*). Ancestral and derived states were defined for all 25 polymorphic chloroplast loci based on allele frequencies estimated across 18 *Oryza* species (Additional file 13: Table S6A and B): ancestral alleles occurred at high frequence (>65 %) in non-AA genome relatives (outgroups) as well as in the *ORSC*, while derived alleles occurred predominantly in the *ORSC* and/or a close relative, i.e. *O. sativa*.

## Additional files

Additional file 1: Table S1. Germplasm information. (XLSX 57 kb)

Additional file 2: Figure S1. Analysis of model complexity (K). (PDF 174 kb)

Additional file 3: Figure S2. Population structure in the *ORSC* and with *O. sativa*. (PDF 362 kb)

Additional file 4: Figure S3. Neighbor Joining (NJ) tree from the *ORSC* based on SNP data. (PDF 232 kb)

Additional file 5: Figure S4. The relationship between geographical and genetic distance of the *ORSC*. (PDF 892 kb)

Additional file 6: Table S2. Pairwise Fst and genetic distance among six *ORSC* and five *O. sativa* subpopulations based on GBS-SNP data. (XLSX 12 kb)

Additional file 7: Table S3. Pericarp and hull color of 157 *ORSC* accessions grouped by subpopulation (at *K* = 6). Number of *ORSC* accessions with hull color and pericarp color phenotypes, grouped by subpopulation. (XLSX 11 kb)

Additional file 8: Table S4 Number of accessions included in *RC* extended haplotype analysis (*ORSC* *N* = 81; *O. sativa* *N* = 405) (XLSX 11 kb)

Additional file 9: Figure S5. Pericarp Color Associated with *RC* Haplotype Groups. (PDF 253 kb)

Additional file 10: Table S5. Chi-square statistic between genetic subgroups and two major traditional species groups, *O. rufipogon* and *O. niva.ra.* **Figure S6. ** Distribution of nuclear subpopulations within traditional species groups in the *ORSC*; *rufipogon* (perennial), *O. nivara* (annual), and *O. spontanea*. (PDF 1329 kb)

Additional file 11: Figure S7. *Geographical distribution of samples based on traditional species nomenclature. (PDF 261 kb)*

Additional file 12: Figure S8. Chloroplast haplotype network including admixed samples. (PDF 598 kb)

Additional file 13: Table S6A *ORSC* chloroplast sequence polymorphisms. **Table S6B**. *Oryza* accessions from Genbank used to define ancestral vs derived mutations across the 25 chloroplast variable sites. (XLSX 18 kb)

Additional file 14: Figure S9. Average DNA sequence diversity (π) within each *ORSC* subpopulation. (PDF 359 kb)

Additional file 15: Table S7. Numeric LD Decay. (XLSX 14 kb)

Additional file 16: Supplemental Text. Extended Materials and Methods (Additional file 18). (DOCX 21 kb)

Additional file 17: Table S8. SNP Information for *RC* Haplotypes. (XLSX 111 kb)

Additional file 18: Figure S10. Distribution of GBS SNPs along the twelve chromosomes of rice. (PDF 119 kb)

## Abbreviations

CI: Confidence interval
*cpGroup*: *Chloroplast group*
GBS: Genotyping-by-sequencing
IRGC: International rice germplasm collection
LD: Linkage disequilibrium
MAC: Minor allele count
NJ: Neighbor joining
*ORSC*: *Oryza rufipogon* *species complex*
W-RDP: Wild rice diversity panel
π: Nucleotide diversity
