# Erratum to: Population dynamics among six major groups of the *Oryza rufipogon* species complex, wild relative of cultivated Asian rice

**DOI:** 10.1186/s12284-017-0156-3

**Published:** 2017-04-26

**Authors:** HyunJung Kim, Janelle Jung, Namrata Singh, Anthony Greenberg, Jeff J. Doyle, Wricha Tyagi, Jong-Wook Chung, Jennifer Kimball, Ruaraidh Sackville Hamilton, Susan R. McCouch

**Affiliations:** 1 Section of Plant Breeding and Genetics, School of Integrative Plant Science, Cornell University, 162 Emerson Hall, Ithaca, NY 14853 USA; 2 TT Chang Genetics Resource Center and International Rice Genebank, International Rice Research Institute, Los Baños, Laguna Philippines; 3 Present Address: Department of Plant Sciences, University of California, Davis, CA 95616 USA; 4 Present Address: School of Crop Improvement, College of PG Studies, Central Agricultural University, Umroi Road, Umiam, Meghalaya India; 5 Present Address: Department of Industrial Plant Science and Technology, Chungbuk National University, Cheongju, Chungubk 28644 Republic of Korea; 6 Present Address: Department of Crop Science, North Carolina State University, Raleigh, NC 27695-762 USA

## Erratum

Following the publication of this article [1], it was brought to our attention that the NCBI Batch ID for the Nuclear GBS dataset was incorrectly cited in the ‘Availability of Data and Materials’ section. The correct NCBI Batch Submission ID is 1062455 (https://www.ncbi.nlm.nih.gov/projects/SNP/snp_viewBatch.cgi?sbid=1062455).

Links to the information are also available at https://ricediversity.org/data/index.cfm

More detailed citations for the Chloroplast SNP data are also now available (https://www.ncbi.nlm.nih.gov/nucleotide). The records can be retrieved from the nucleotide database by searching Entrez Nucleotide using the accession numbers below:

* chloroplast_region1: KX556608 - KX556920

* chloroplast_region2: KX556921 - KX557233

* chloroplast_region3: KX556295 - KX556607

* chloroplast_region4: KX555982 - KX556294
