# Supplementary material for: Population Dynamics Among six Major Groups of the Oryza rufipogon Species Complex, Wild Relative of Cultivated Asian Rice
Source: Rice (N Y). 2016 Oct 12;9:56. doi: 10.1186/s12284-016-0119-0 (PMC5059230; doi:10.1186/s12284-016-0119-0)
Supplement: Supplementary file 2 — Analysis of model complexity (K). (PDF 174 kb) [file 12284_2016_119_MOESM2_ESM.pdf]

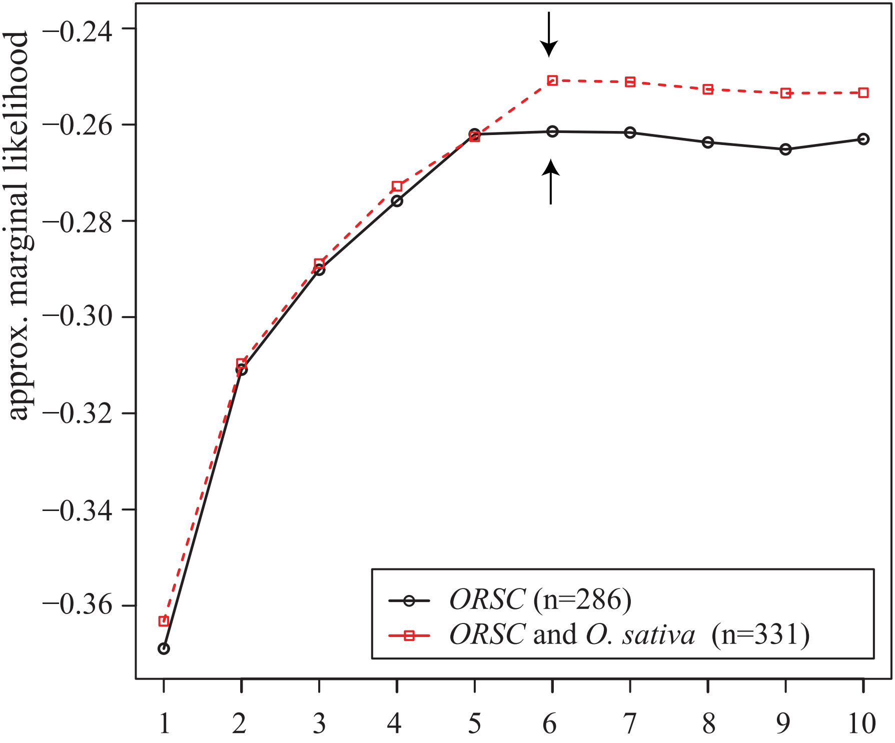

Figure S1. Analysis of model complexity (K). K value evaluated based on approximations of the marginal likelihood of the data computed by *fastStructure* among the *ORSC* and the *ORSC* with *O. sativa*; black arrows indicate the highest value of marginal likelihood.
