# Supplementary material for: Population Dynamics Among six Major Groups of the Oryza rufipogon Species Complex, Wild Relative of Cultivated Asian Rice
Source: Rice (N Y). 2016 Oct 12;9:56. doi: 10.1186/s12284-016-0119-0 (PMC5059230; doi:10.1186/s12284-016-0119-0)
Supplement: Supplementary file 3 — Population structure in the ORSC and with O. sativa. (PDF 362 kb) [file 12284_2016_119_MOESM3_ESM.pdf]

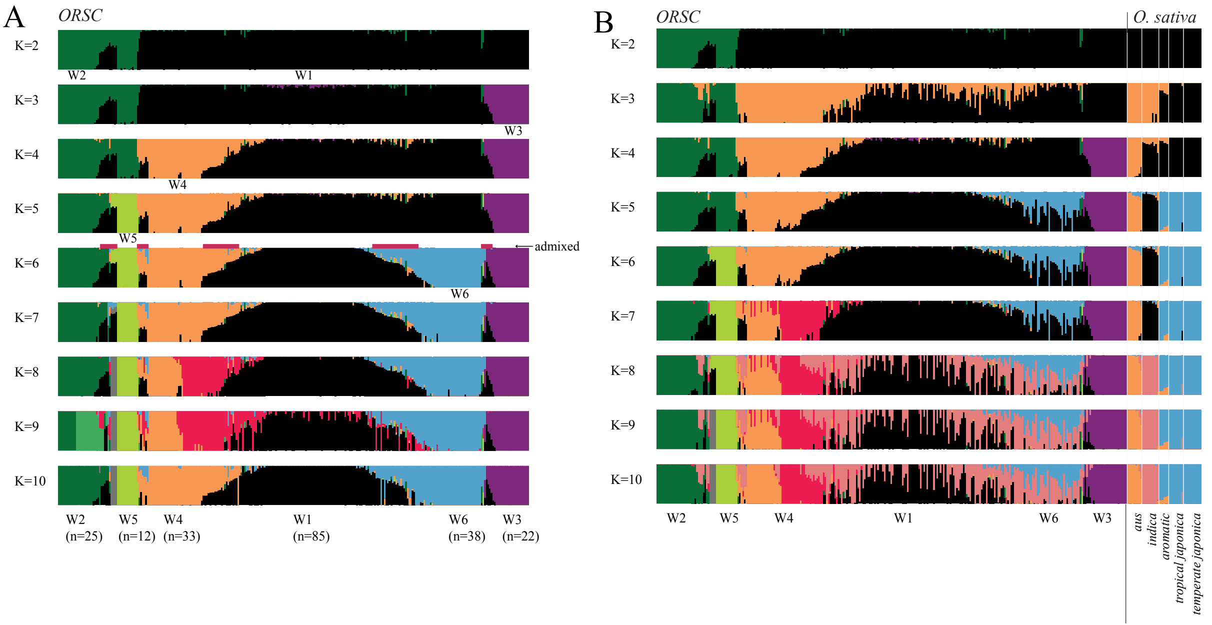

Figure S2. Population structure in the *ORSC* and with *O. sativa*. (A) *fastStructure* analysis for 286 *ORSC* samples from K=2 to K=10 based on 113,996 SNPs; subpopulation identity defined at K=6 (see Fig. 1A); wild group numbers, W1-W6, correspond to order of divergence; admixed accessions indicated by red rectangles above K=6 panel. (B) *fastStructure* analysis for 286 *ORSC* samples and 45 *O. sativa* accessions from K=2 to K=10 based on same 113,996 SNPs; the three *O. sativa* subpopulations correspond to *aus* (orange), *indica* (pink) and *japonica* (blue).
