# Supplementary material for: Population Dynamics Among six Major Groups of the Oryza rufipogon Species Complex, Wild Relative of Cultivated Asian Rice
Source: Rice (N Y). 2016 Oct 12;9:56. doi: 10.1186/s12284-016-0119-0 (PMC5059230; doi:10.1186/s12284-016-0119-0)
Supplement: Supplementary file 4 — Neighbor Joining (NJ) tree from the ORSC based on SNP data. (PDF 232 kb) [file 12284_2016_119_MOESM4_ESM.pdf]

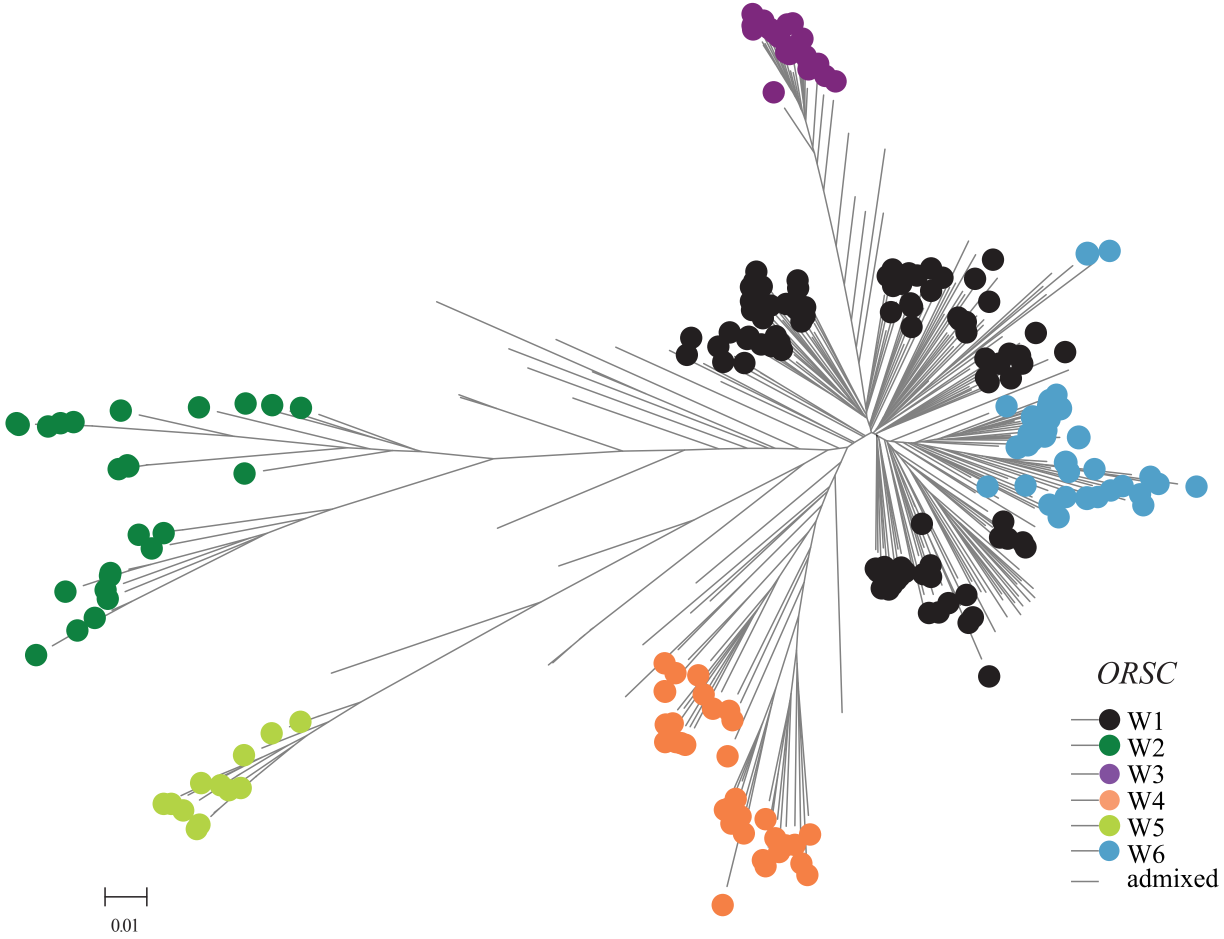

Figure S3. Neighbor Joining (NJ) tree from the *ORSC* based on SNP data. Circle color corresponds to subpopulation identity as in Fig. 1A.
