# Supplementary material for: Population Dynamics Among six Major Groups of the Oryza rufipogon Species Complex, Wild Relative of Cultivated Asian Rice
Source: Rice (N Y). 2016 Oct 12;9:56. doi: 10.1186/s12284-016-0119-0 (PMC5059230; doi:10.1186/s12284-016-0119-0)
Supplement: Supplementary file 5 — The relationship between geographical and genetic distance of the ORSC. (PDF 892 kb) [file 12284_2016_119_MOESM5_ESM.pdf]

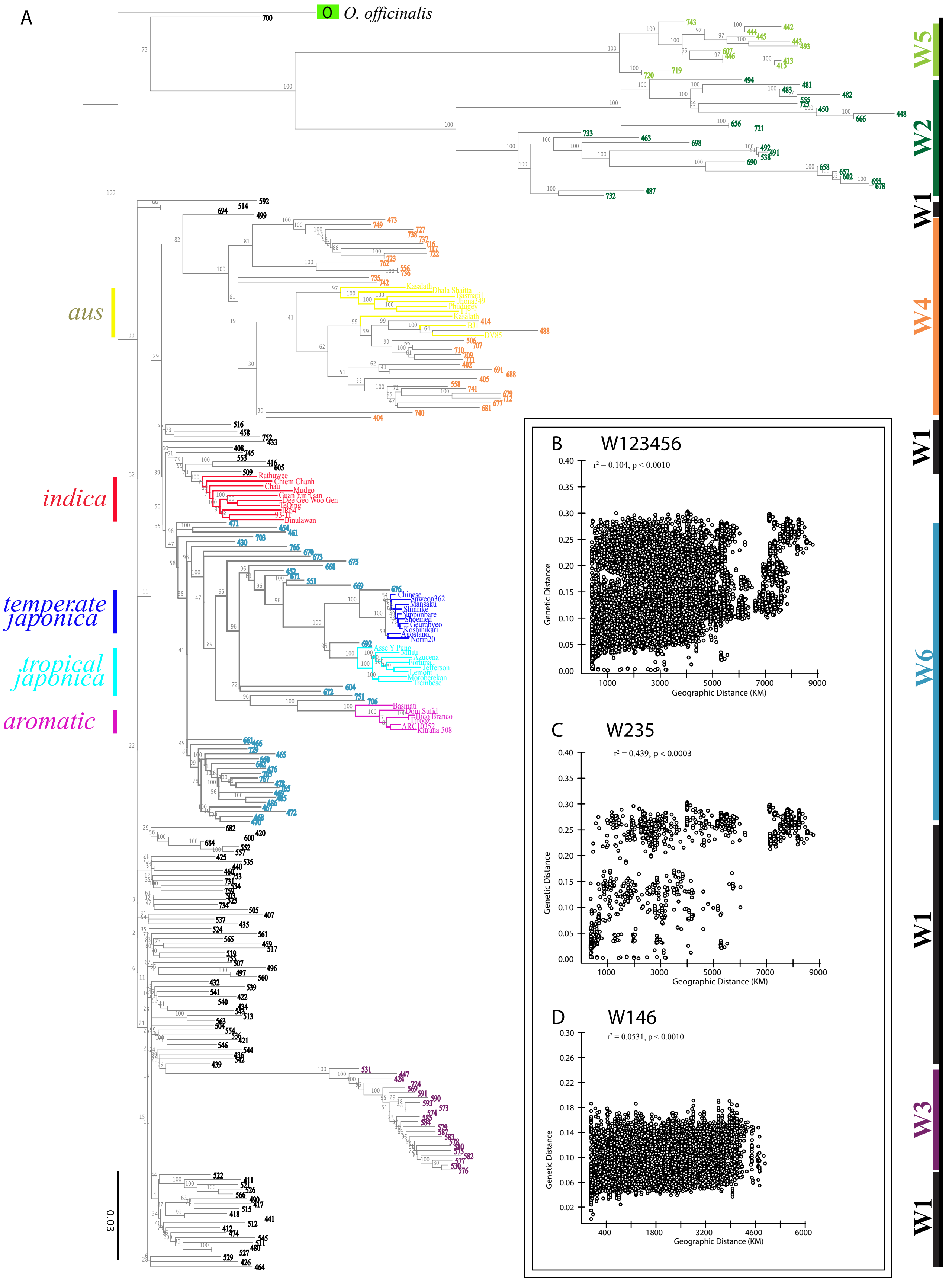

Figure S4. The relationship between geographical and genetic distance of the *ORSC*. Consensus Neighbor Joining (NJ) dendrogram of 262 individuals (215 wild accessions of groups W1-W6, 45 *O. sativa* accessions, and one *O. officinalis* outgroup) based on 100 bootstrap replications. Colors indicate subpopulation identity; colored bars on left correspond to *O. sativa* subpopulation groups; colored bars on right correspond to *ORSC* subpopulations. Right panel graphs show results of the Mantel test between genetic distance and geographical distance for individuals in: all six *ORSC* subpopulations (S4B); the three independent *ORSC* subpopulations (S4C); and the three *O. sativa*-like *ORSC* subpopulations (S4D).
