# Supplementary material for: Population Dynamics Among six Major Groups of the Oryza rufipogon Species Complex, Wild Relative of Cultivated Asian Rice
Source: Rice (N Y). 2016 Oct 12;9:56. doi: 10.1186/s12284-016-0119-0 (PMC5059230; doi:10.1186/s12284-016-0119-0)
Supplement: Supplementary file 9 — Pericarp Color Associated with RC Haplotype Groups. (PDF 253 kb) [file 12284_2016_119_MOESM9_ESM.pdf]

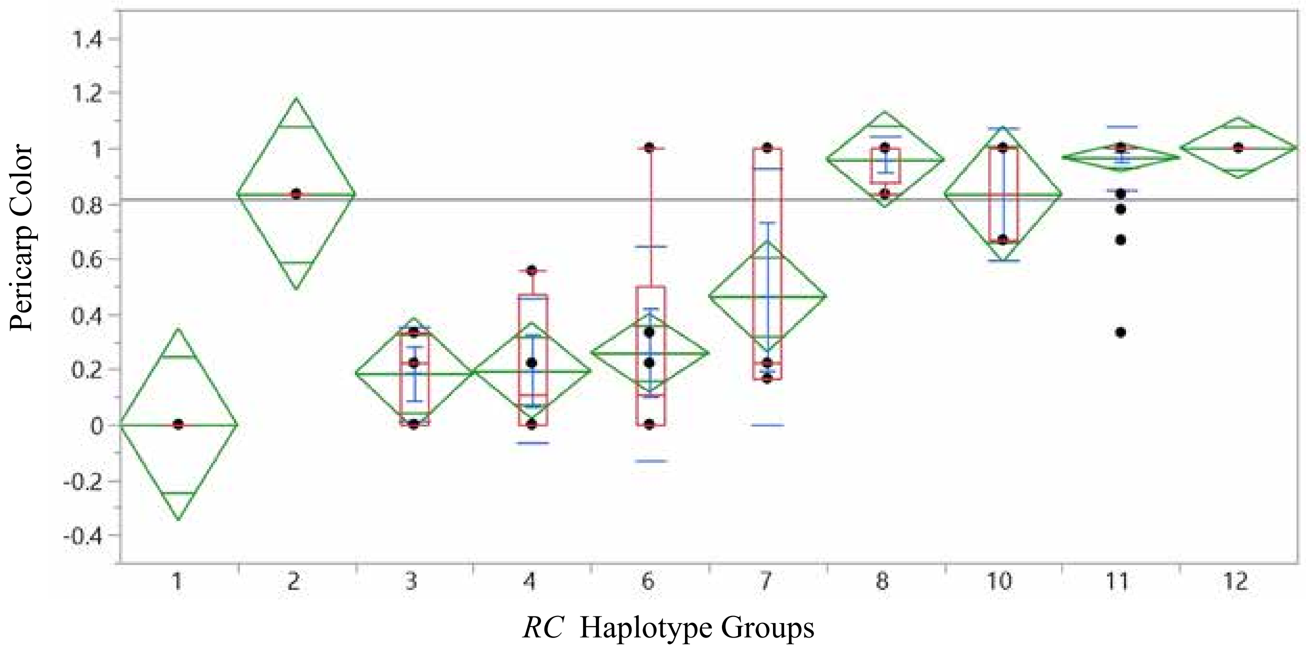

Figure S5. Pericarp Color Associated with *RC* Haplotype Groups. Quantile boxplots showing the pericarp color of 81 accessions across 12 *RC* extended haplotype groups. Points of green diamonds indicate upper and lower 95% confidence intervals. Mean error and standard deviations are indicated by the inner and outer pairs of blue horizontal lines, respectively. Pericarp color was scored as: red pericarp=1.0; white pericarp=0, evaluated on three seeds each, produced by three individuals from each accession.
