# Supplementary material for: Population Dynamics Among six Major Groups of the Oryza rufipogon Species Complex, Wild Relative of Cultivated Asian Rice
Source: Rice (N Y). 2016 Oct 12;9:56. doi: 10.1186/s12284-016-0119-0 (PMC5059230; doi:10.1186/s12284-016-0119-0)

Table S5. Chi-square statistic between genetic subgroups and two major traditional species groups, *O. rufipogon* and *O. nivara*

Subpopulation	Species					Total
	O. rufipogon	*O. nivara*	*O. spontanea*	*O. rufipogon* x *O. nivara* or *O. nivara* x *O. rufipogon*	*O. rufipogon* x *O. sativa* or *O. sativa* x *O. rufipogon*	
W1	77	1	4	2	1	85
W2	9	14	0	2	0	25
W3	22	0	0	0	0	22
W4	7	21	3	2	0	33
W5	0	10	2	0	0	12
W6	19	4	9	6	0	38
admix	37	22	1	9	2	71
SUM	171	72	19	21	3	286
						286

Tests

N	DF	-LogLike	Rsquare
184	5	60.519449	0.5623

Test	ChiSquare	Prob>ChiSq
Likelihood Ratio	121.039	<.0001*
Pearson	108.103	<.0001*

Figure S6: Distribution of nuclear subpopulations within traditional species groups in the *ORSC*; *rufipogon* (perennial), *O. nivara* (annual), and *O. spontanea*.

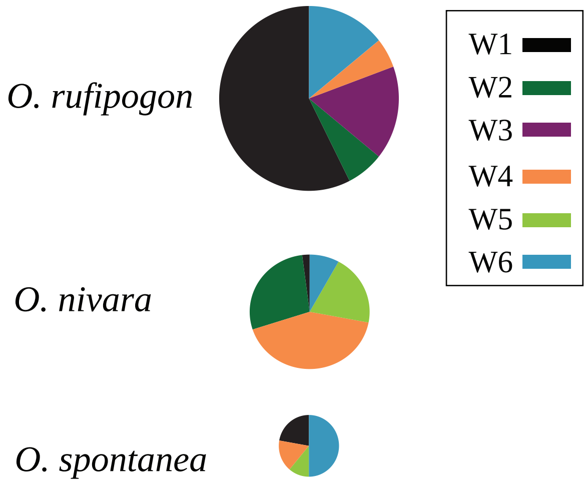

Supplement: Supplementary file 10 — Chi-square statistic between genetic subgroups and two major traditional species groups, O. rufipogon and O. niva.ra. Figure S6. Distribution of nuclear subpopulations within traditional species groups in the ORSC; rufipogon (perennial), O. nivara (annual), and O. spontanea. (PDF 1329 kb) [file 12284_2016_119_MOESM10_ESM.pdf]
