# Supplementary material for: Population Dynamics Among six Major Groups of the Oryza rufipogon Species Complex, Wild Relative of Cultivated Asian Rice
Source: Rice (N Y). 2016 Oct 12;9:56. doi: 10.1186/s12284-016-0119-0 (PMC5059230; doi:10.1186/s12284-016-0119-0)
Supplement: Supplementary file 11 — Geographical distribution of samples based on traditional species nomenclature. (PDF 261 kb) [file 12284_2016_119_MOESM11_ESM.pdf]

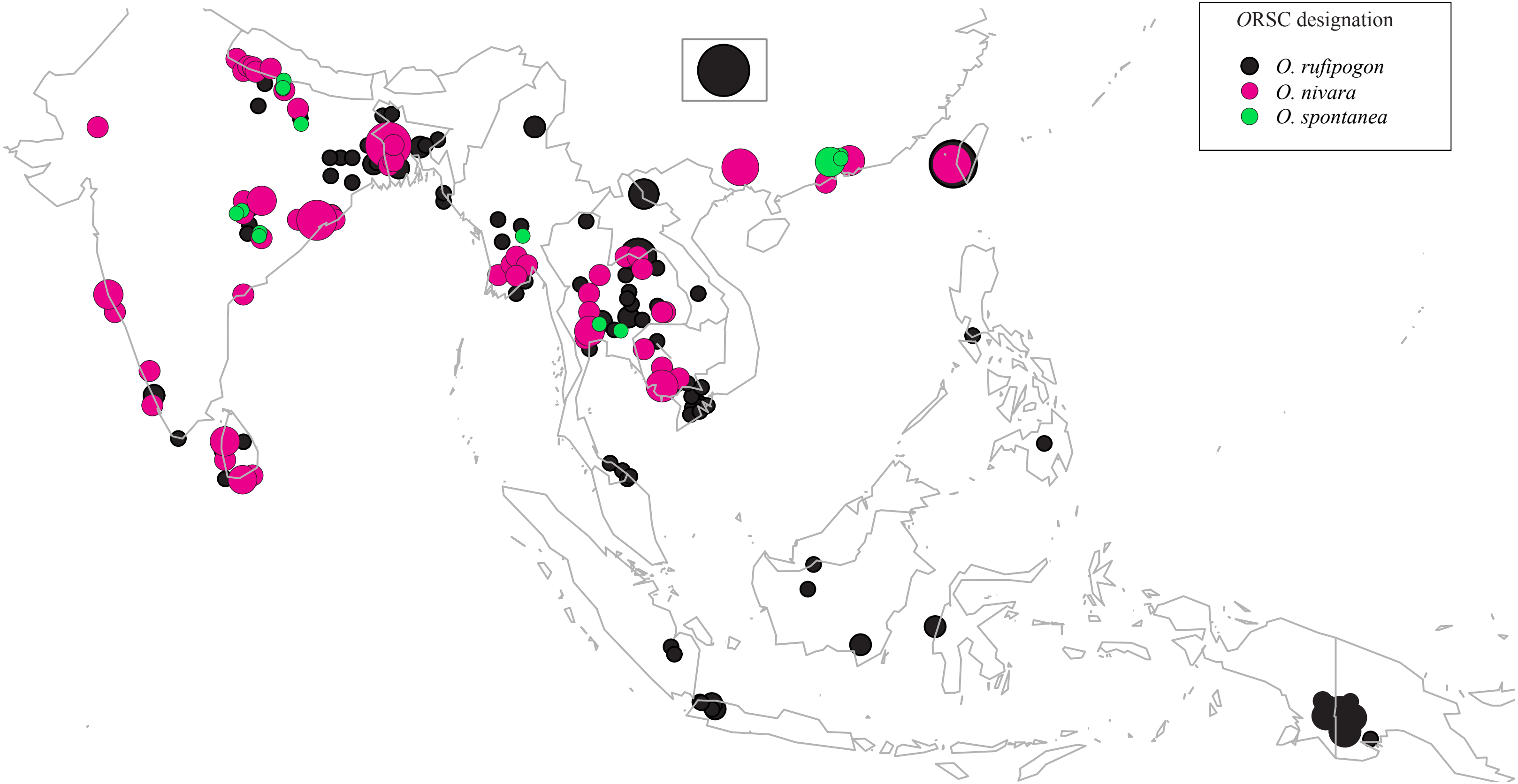

Figure S7: Geographical distribution of samples based on traditional species nomenclature. Geographical map showing distribution of samples from each species designation where size of circle corresponds to relative number of samples; Chinese accessions lacking location detail indicated in closed rectangle.
