# Supplementary material for: Population Dynamics Among six Major Groups of the Oryza rufipogon Species Complex, Wild Relative of Cultivated Asian Rice
Source: Rice (N Y). 2016 Oct 12;9:56. doi: 10.1186/s12284-016-0119-0 (PMC5059230; doi:10.1186/s12284-016-0119-0)
Supplement: Supplementary file 12 — Chloroplast haplotype network including admixed samples. (PDF 598 kb) [file 12284_2016_119_MOESM12_ESM.pdf]

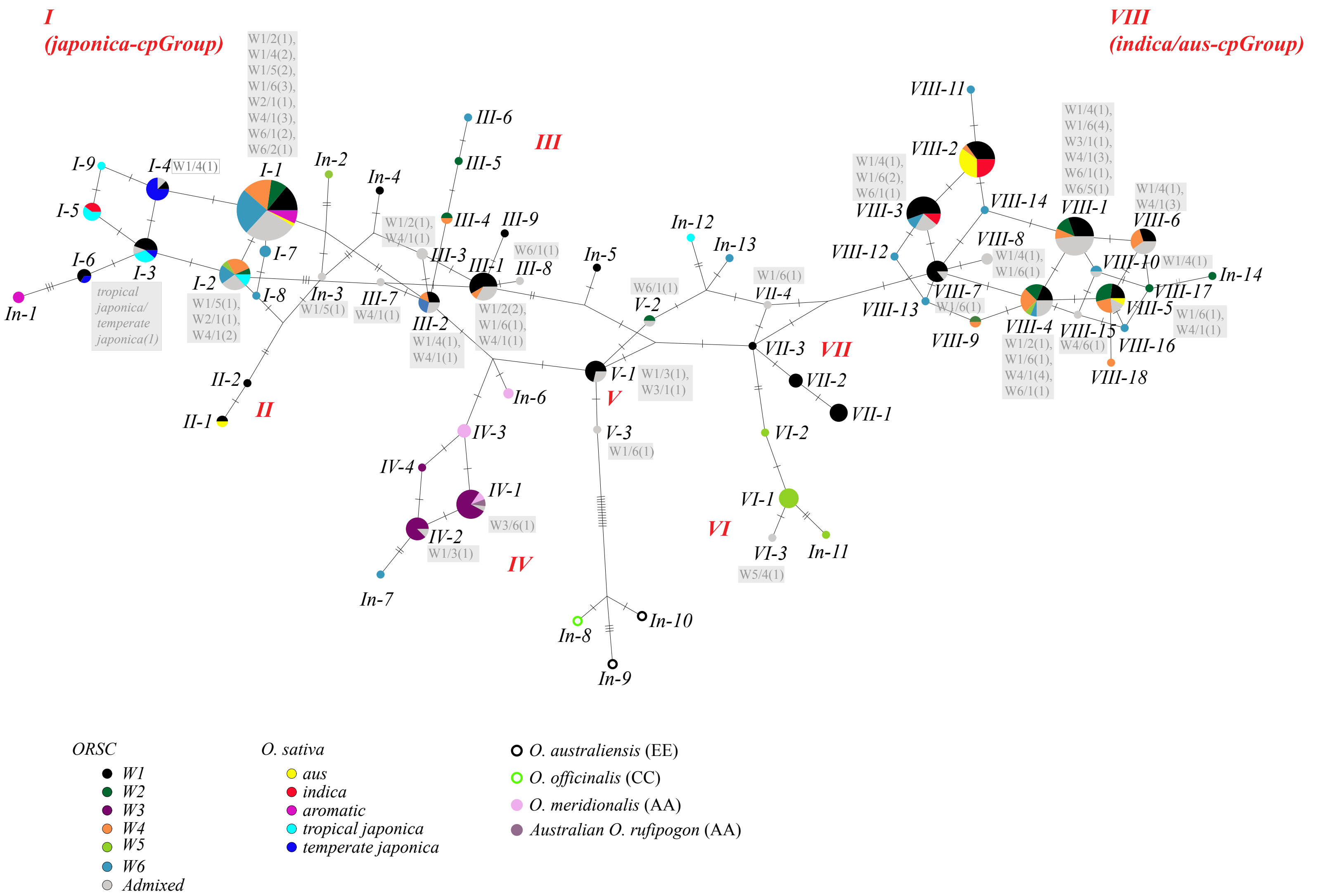

Figure S8. Chloroplast haplotype network including admixed samples. (A) Haplotype network for the *ORSC* and *O. sativa* samples based on 25 chloroplast variants; single mutations indicated as hatches between haplotypes; chloroplast groups (*cpGroup*) *I* to *VIII* indicated in rectangles; size of nodes (circles) is proportional to haplotype frequency; colors indicate proportion of individuals from each subpopulation (based on GBS data at K=6 in Fig. 1A) that carry the haplotype; text highlighted in gray indicates admixed accessions with genetic information.
