# Supplementary material for: Population Dynamics Among six Major Groups of the Oryza rufipogon Species Complex, Wild Relative of Cultivated Asian Rice
Source: Rice (N Y). 2016 Oct 12;9:56. doi: 10.1186/s12284-016-0119-0 (PMC5059230; doi:10.1186/s12284-016-0119-0)
Supplement: Supplementary file 14 — Average DNA sequence diversity (π) within each ORSC subpopulation. (PDF 359 kb) [file 12284_2016_119_MOESM14_ESM.pdf]

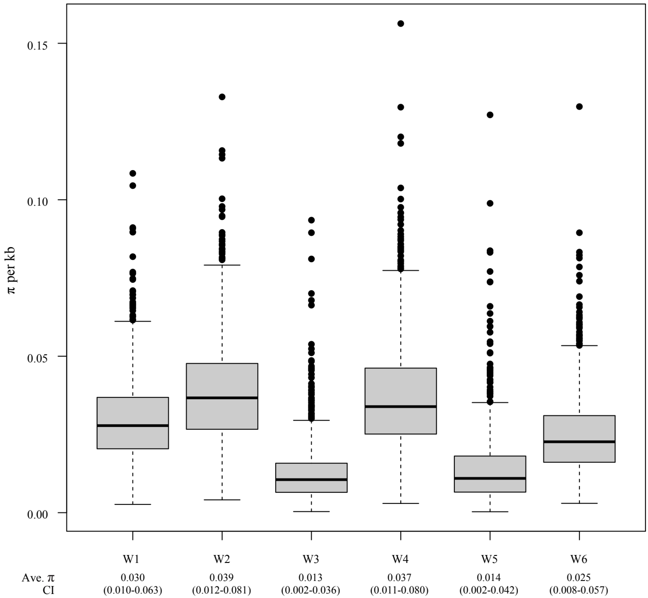

Figure S9. Average DNA sequence diversity (π) within each *ORSC* subpopulation. Average π estimated based on pairwise sequence diversity; CI = 95% confidence interval. Note: These values represent underestimates by roughly one to two orders of magnitude compared to full sequencing data because only a subset of the DNA between any pair of SNPs was actually sequenced in our GBS data set. While they are not expected to be consistent with data sets that are based on complete sequences, our estimates are useful for among-poulation comparisons within our data set.
