# Supplementary material for: Population Dynamics Among six Major Groups of the Oryza rufipogon Species Complex, Wild Relative of Cultivated Asian Rice
Source: Rice (N Y). 2016 Oct 12;9:56. doi: 10.1186/s12284-016-0119-0 (PMC5059230; doi:10.1186/s12284-016-0119-0)
Supplement: Supplementary file 16 — Supplemental Text. Extended Materials and Methods (Additional file 18). (DOCX 21 kb) [file 12284_2016_119_MOESM16_ESM.docx]

# Supplemental Text: Extended Materials and Methods

# Germplasm

A total of 286 wild accessions originated in 15 different countries and were selected to represent the geographic range of *O. rufipogon* using available passport data. Based on this information, the largest number of accessions was classified as *O. rufipogon* (N=171)*,* while 72 accessions were classified as *O. nivara* and 19 as *O. spontanea.* Others were classified as species mixtures, as indicated in Additional File 1: Table S1 and Additional File 10: Table S5. An accession of *O. officinalis* (IRGC 105220, CC genome) served as an outgroup to help differentiate between ancestral and derived alleles. Fifty accessions of *O. sativa,* selected as representative of the five major subpopulations of *O. sativa,* served as controls for evaluating the relationship between wild and cultivated accessions: these included 10 *aus*, 11 *indica,* 7 *aromatic*, 11 *tropical japonica*, 10 *temperate japonica* and 1 *temperate/tropical japonica*  admixed variety from the Rice Diversity Panel 1 (RDP1) (Additional File 1: Table S1B) (Eizenga et al, 2014)*.*

***Plant Grow-out***

*O. rufipogon* accessions were grown in 6” pots under greenhouse conditions (85 °F day/ 75 °F night, 11 hour light cycle (7am-6pm), 81-85% humidity; Guterman Bioclimatic Laboratory and Greenhouse Complex, Cornell University, Ithaca, NY) during 2006-2007. Prior to planting, wild seeds were cold treated at -20 °C for 48 hours. After returning to room temperature, seeds were surface-sterilized with a 30-second soak in 70% EtOH, followed by a 15-minute soak in a 20% bleach solution, and three washes with ddH_2_O. Seeds were planted individually in 15.24cm clay pots, 1 cm below the soil surface in damp Cornell mix (0.107 m^3^ of Peat Moss: 9.07 kg of Vermiculite: 4.53 kg of Perlite: 1.13 kg of Pulverized Dolomitic Limestone: 0.91 kg of Jack's Media Mix Plus III (10-5-10 (N-P-K)). Pots were arranged on greenhouse benches in a randomized block design. Throughout the growing period, standing water was maintained in tanks at a constant depth of 8-10 cm. Plants were fertilized twice per week with 300 ppm Nitrogen, Jacks 15-5-15 Cal Mag, 30 ppm Iron, and Sprint 330 Iron Chelate. Panicles from individual plants were covered with glassine or waxed paper pollination bags prior to stigma exertion to prevent cross-pollination and shattering and to facilitate the collection of self-pollinated (S_1_-S_3_) seed.

# Genotyping

Genotyping-By-Sequencing: The six GBS libraries were prepared as described in Spindel et al. (2013). They contained samples of 286 *O. rufipogon*, one *O. officinalis* and 45 *O. sativa* accessions, of which 18 *O. rufipogon* and two *O. sativa* accessions were replicated both within and between plates to compare SNP calls among genetically identical samples. The six GBS libraries produced an average of 247,501,781 reads per library and contained an average of 128,400,227 QC-passed barcoded reads. A total of 5,591,115 tags were identified and aligned across the six libraries; 3,604,943 (64.5%) tags were uniquely aligned, 1,173,766 (21%) tags were aligned to more than one position in the genome; 812,406 (14.5%) tags were not aligned.

GBS libraries were sequenced using Illumina HiSeq 2500 with short reads (<100 bp and single-end reads) at the Biotechnology Resource Center (BRC) Genomics Facility at Cornell University. The Tassel 3 GBS Plugin was used to call and filter SNPs (Glaubitz et al 2014). Barcoded Fastq files were read with the FastqToTqgCountPlugin to generate tag counts and merged with the MergeMultipleTagCountPlugin (option: -c 5). The sequence tags were aligned to the Nipponbare reference genome (MSU v7) using Bowtie2 (Langmead and Salzberg 2012) and converted to a SAM format and a binary tagsOnPhysicalMap file for subsequent processes. Tag information of each taxa for every fastq file was generated and merged with the FastqToTBTPlugin (option: -c 5) and the MergeTagsByTaxaFilesPlugin, respectively. After alignment of tags, SNPs were called and filtered through the TagsToSNPByAlignmentPlugin (option: -mnMAF 0.01 -mnMAC 10) and the MergeDuplicateSNPsPlugin (option: -misMat 0.05). SNP genotype data was generated as a hapmap file. This SNP dataset was filtered with call rates greater than 50% per SNP locus with MAC 3 using Plink for *ORSC* and *O. sativa* independently, as well as *ORSC* and *O. sativa* together.

When we applied the same method to call SNPs for *O. rufipogon* and *O. sativa* samples together, a total of 119,393 SNPs met these criteria, but the set of 113,739 SNPs was preferred for this study because the *ORSC* was the major focus. The 113,739 SNPs were well-distributed throughout the rice genome, with pericentromeric regions being the most sparse (Additional File 18: Fig. S10). The average genotype call rate per accession for *ORSC* samples was 72%. A total of 38,270 SNPs were selected for *O. sativa* and used to define subpopulation groups (Additional File 1: Table S1). To evaluate SNP calling accuracy, SNP genotype concordance was calculated using a set of 18 replicated *O. rufipogon* accessions and two *O. sativa* accessions; an average of 99.33% concordance within plates and 98.98% concordance between plates was observed.

**References**

1. Eizenga, G. C. *et al.* Registration of the Rice Diversity Panel 1 for Genomewide Association Studies. *J. Plant Regist.* **8,** 109–116 (2014).

2. Spindel, J. *et al.* Bridging the genotyping gap: using genotyping by sequencing (GBS) to add high-density SNP markers and new value to traditional bi-parental mapping and breeding populations. *Theor. Appl. Genet.* **126,** 2699–716 (2013).

3. Glaubitz, J. C. *et al.* TASSEL-GBS: a high capacity genotyping by sequencing analysis pipeline. *PLoS One* **9,** e90346 (2014).

4. Langmead, B. & Salzberg, S. L. Fast gapped-read alignment with Bowtie 2. *Nat. Methods* **9,** 357–359 (2012).
