# Supplementary material for: Population Dynamics Among six Major Groups of the Oryza rufipogon Species Complex, Wild Relative of Cultivated Asian Rice
Source: Rice (N Y). 2016 Oct 12;9:56. doi: 10.1186/s12284-016-0119-0 (PMC5059230; doi:10.1186/s12284-016-0119-0)
Supplement: Supplementary file 18 — Distribution of GBS SNPs along the twelve chromosomes of rice. (PDF 119 kb) [file 12284_2016_119_MOESM18_ESM.pdf]

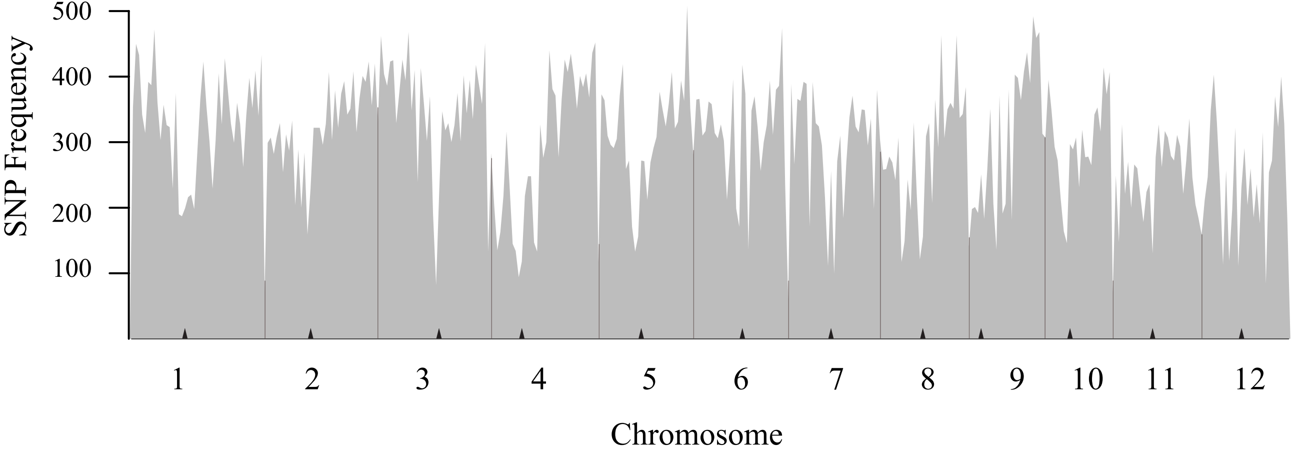

Figure S10. Distribution of GBS SNPs along the twelve chromosomes of rice. SNP frequency calculated in 1Mb window; black triangles indicate centromere positions.
